# The impact of school life and family involvement on Western China junior high school students’ wellbeing at multidimensional levels

**DOI:** 10.1186/s13690-022-00863-w

**Published:** 2022-04-02

**Authors:** Yipeng Lv, Ye Gao, Bihan Tang, Fan Cheng, Zeqi Chen, Jing Wu, Hongyang Yang, Xu Liu

**Affiliations:** 1 School of Public Health, School of Medicine, Shanghai Jiao Tong University, Shanghai, People’s Republic of China; 2 Department of Gastroenterology, First Affiliated Hospital, Naval Medical University of the Chinese People’s Liberation Army, Shanghai, People’s Republic of China; 3 Department of Health Service, College of Health Service, Naval Medical University of the Chinese People’s Liberation Army, Shanghai, People’s Republic of China; 4 Department of Endodontics, School and Hospital of Stomatology, Tongji University, Shanghai Engineering Research Center of Tooth Restoration and Regeneration, Shanghai, People’s Republic of China; 5 School of nursing, Naval Medical University of the Chinese People’s Liberation Army, Shanghai, People’s Republic of China; 6 Department of Blood Trans fusion, Naval Medical Center of the Chinese People’s Liberation Army, Shanghai, People’s Republic of China; 7 President’s Office, Xinhua Hospital Affiliated to Shanghai Jiao Tong University School of Medicine, Shanghai, People’s Republic of China

**Keywords:** Junior high school students, Subjective health, Pediatric patient-reported outcomes measurement information system (PROMIS), BMI

## Abstract

**Background:**

The focus of students’ health concerns has gradually progressed from the single factor of physical health to comprehensive health factors, and the physical and mental health of students are now generally considered together. This study focuses on exploring the status of junior high school students’ physical health and their subjective health assessment with the major societal factors that affect students’ lives: School Life and Family involvement. In addition, we explore the main factors influencing students’ subjective health.

**Methods:**

A cross-sectional survey was conducted with 190 Tibetan junior high school students in the Maozhuang Township. The intentional sampling was used to choose the research object. The structured questionnaire comprised four parts, namely social and demographic information, family condition, school life, and subjective health quality which was assessed by PROMIS (Chinese version of the Pediatric Patient-Reported Outcomes Measurement Information System).

**Result:**

The average height and weight of boys and girls are statistically different (*p*-values of 0.026 and 0.044, respectively), but there is no statistically significant difference in BMI (Body Mass Index) between boys and girls (p-value of 0.194). The average values of the five dimensions of depression, anger, anxiety, fatigue, and peer relationships in the PROMIS of the research subjects were 58.9 ± 5.3, 53.3 ± 8.0, 58.1 ± 7.3, 52.8 ± 8.0, 39.3 ± 6.6. In the demographic dimension, the grade was the main factor influencing anger (*p* < 0.01) and fatigue (*p* < 0.01), while gender was related to peer relationships (*p* = 0.02). In the family dimension, the father’s educational level was related to peer relationships (*p* = 0.05), while the family financial situation was related to depression (*p* = 0.01). In the school life dimension, relationship with classmates was found to affect anger (*p =* 0.05), while homework was related to anxiety (*p* = 0.02) and fatigue (*p =* 0.05).

**Conclusion:**

the physical health index BMI and subjective health evaluation of students are worse than students of more developed areas in China. Their family environment and school life all have varying degrees of impact on the five subjective health outcomes. There are differences in gender and grade level. The government and society need to pay more attention to the physical and mental health of students in remote and underdeveloped areas and improve their health through a student nutrition plan and the establishment of mental health offices.

## Background

Modern health comprises physical, psychological, and social aspects. The interaction of various elements of individual health together constitutes the dynamic balance of individual healt h[1]. Much of the current research on the health of students describes their mental and physical health, along with their mutual influenc e[2]. Scholars have found that the mental health problems of students exert a continuous influence on their future live s[3, 4]. However, there are relatively few evaluations of the subjective health status of students compared with physical examinatio n[5]. There are also few health protection policies (such as health insurance) for this age group, compared with people that are older. Carrying out research that monitors junior high school students’ subjective health and the factors influencing it can help detect early risk factors in the growth and development of students with limited health resources available to them. This type of research can justify interventions to improve their physical and mental health, which would be highly beneficial initiative to society as a whole.

With their social development, young people are exposed to an increasing number of health risk factors, especially the emotional aspects of their fast-paced life. In response, and multi-angle and continuous health care is being adopted by various organizations. The focus of students’ health concerns has gradually progressed from the single factor of physical health to comprehensive health factors, and the physical and mental health of students are now generally considered together. In China, however, there are still great regional differences in the degree of attention given to adolescent health. Specifically, more studies are focused on the physical and mental health of students in relatively developed region s[5]. At present, physical examination for students is generally carried out in various regions of China, and much attention is being paid to their physical health conditions. However, in a study that assessed Chinese students’ mental health, only 66% of students in the sample had a passing score in maintaining mental healt h[6]. In the field of psychology, there are numerous studies on the mental health of specific groups of students in urban areas or on the mental health status of people such as students or migrants during a specific time period. These include studies on the mental health of students during the Covid-19 pandemic, or before and after their parents move to a large cit y[7–13]. At present, there is insufficient research on the physical and mental health of people in underdeveloped areas in western China, especially ethnic minority youth. Social concern is generally lower for this group, and they are more vulnerable to psychological risk factors that come from society or the family. Impacted by a diversified society, current students are inundated with various sources of information about the outside world and about the relatively backward development of their regions, which inevitably produces a great confusion in their minds. The corresponding risk factors of whether this will affect the mental health status, and later the physical and social health status of those students, are still not unknow n[5, 14].

At present, the studies on the subjective health of students are mostly focused on subjective health complaints and health literac y[15–17]. Health-related quality of life (HRQoL) is often used as an indicator in subjective health evaluations. In particular, mental health research focuses on the evaluation of depression and anxiet y[18, 19]. Depression is used as an indicator of mental health by researchers analyzing depression and anxiety among students with different characteristics and different influencing factor s[20–23]. However, quality of life (QOL) only reflects the status of the physical component summary scale (PCS) and the mental component summary scale (MCS), while ignoring other dimensions such as social relations. The Pediatric Patient-Reported Outcomes Measurement Information System (PROMIS) is a set of person-centered measures that evaluate and monitor physical, mental, and social health in adults and children. It can also be used with the general population [24]. The benefit of using PROMIS is similar comparisons between different populations can be conducted to compare the subjective health of people with different types of diseases. This is especially useful for assessing students with chronic disease s[15, 16, 25]. This scale has been widely used in the subjective health evaluation of children with different diseases, for analyzing the different health dimensions of patients, and for the implementing targeted interventions to improve the effectiveness of diagnosis and treatmen t[26, 27]. At present, the PROMIS questionnaire is insufficiently applied in the field of subjective health for normal students. However, it can be a useful tool for early detection of adolescent health problems so that timely intervention can be mad e[28].

This study was carried out in Nangqian County, in the Yushu Prefecture of the Qinghai Province in China. It is located in the eastern part of the Qinghai-Tibet Plateau, with an average elevation of more than 4000 m. It is a semi-agricultural and semi-pastoral area dominated by animal husbandry and supplemented by agriculture. Tibetans account for over 99.4% of the total population. This region is in the economically underdeveloped area of western China. With the help of various poverty alleviation projects initiated by the Chinese government, it was officially announced by the government on April 21, 2020, that Nangqian County had withdrawn from the list of the poverty-stricken counties. This study focuses on the underdeveloped ethnic minority areas in western China. We specifically explore the status of junior high school students’ physical health (including BMI, Physical examination results and so on) and their subjective health assessment with PROMIS questionnaire including depression, anxiety, eta, as mentioned above. We analyze the major societal factors that affect students’ lives: School Life and Family involvement. In addition, we explore the main factors influencing students’ subjective health. This can provide a reference for evidence-based decision making and early intervention and improvement of adolescent health by outlining their health risk factors at an early stage.

## Methods

### Study design and participants

A cross-sectional survey was conducted in August 2019 with 190 Tibetan junior high school students from Zirong junior high school located in Maozhuang Township, Nangqian County, Qinghai Province. This is a Tibetan ghetto area located in the Qinghai-Tibet Plateau. The environmental and socioeconomic condition of Nangqian County is representative of minority areas in Western China, which are usually characterized by high altitude, cold climate, animal husbandry, and traffic inconvenience. Since western China is a sparsely-populated area which make the random sampling process quite difficult and impractical, the intentional sampling based on experts was used and the representation, scientificalness and convenience were fully concerned in the research. We select Zirong junior high school in 29 junior high schools in Nangqian County because this school locates near the border of Qinghai and Tibet Province and mainly enrolls adolescent of herdsman family from 3 townships in Nangqian, Qinghai and 1 township in Changdu, Tibet. Such diversity of origins of students could improve the representativeness of sampling. The Zirong junior high school is a boarding school, and students typically return home once or twice a month. All eligible students in Zirong junior high school were invited to participate in the survey. Students in grades three through six were included in the study. Students were excluded if they suffered from chronic disease, acute medical conditions in the recent 2 weeks, or refused to cooperate. Of the 433 students in Zirong Junior high school, 250 students were in grades three through six and met the inclusion criterion. Our final sample included 190 students who completed the entire survey.

### Data collection

The survey was conducted by eight undergraduate students from the Navy Military Medical University. The project leader conducted a centralized training in Shanghai for the eight investigators before the initiation of the research. He briefed the students about the details of the study and answered any questions they had. The investigation was conducted between August 15-25, 2019. The survey was conducted in the classrooms of the Zirong Junior high school. The researchers briefed the respondents about the details of the questionnaire and answered relevant questions.

Permission for conducting this study was obtained from the school principal, who made an announcement about our research to the students and their parents with their consent. We also obtained approval from the ethics committee of the Naval Medical University, and all participants voluntarily agreed to participate in our survey.

### Measurements

The structured questionnaire comprised four parts, namely social and demographic information, family condition, school life, and subjective health quality. The collected social and demographic information included age, grade, sex, ethnicity, and religious faith. Inquiries about family condition included students’ residential area, whether they lived with parents or not, number of household members, parents’ educational level, and household income. Choices of residential area included urban, agricultural, or pastoral areas. Students “not living with parents” refers to the ones that were left behind adolescent (i.e., the biological parents went to the city to work, leaving the child behind to stay with grandparents or other living relatives). Inquiries about school life included number of roommates, frequency of adolescent’s visits home, distance from school to home, daily hours spent on assignments, and daily sleeping hours. Choices for the frequency of visits home included four times per month, two to three times per month, once a month, and less than once a month. Home distances were measured by the number of hours adolescent spent returning home, and the choices included less than 1 hour, two to 4 hours, four to 6 hours, and longer than 6 hours.

The students’ subjective quality of life assessment was measured using the Chinese version of the Pediatric Patient-Reported Outcomes Measurement Information System (PROMIS), which has previously been validated among Chinese children with tumors. PROMIS short forms, including Pediatric v1.1 - Depressive Symptoms 8b, Pediatric v1.0 - Anger 6a, Pediatric v1.1 - Anxiety 8b, Pediatric v1.0 - Fatigue 10a, and Pediatric v1.0 - Peer Relationships 8a were completed by each participant. These five domains were selected by expert consensus from 21 domains of the pediatric PROMIS. Short forms include eight items for all domains except fatigue (ten items) and anger (six items). All of these PROMIS items used the context statement “In the past 7 days.” Responses included five options ranging from “never” to “almost always”. The raw score and the T score of each short form was calculated in accordance with the score manual available at http://www.nihpromis.org. Each PROMIS pediatric domain generated a T-score with a mean of 50 and a standard deviation of ten. The mean of 50 reflects the calibration population and does not represent the general population or other specific group. Higher scores indicate higher measured symptoms, thus signifying worse symptoms of depression, anxiety, fatigue, and anger but meaning better condition for peer relationships. PROMIS pediatric measures have consistently achieved a reliability of 0.85 or greater over a range of two to four standard deviations with the short forms.

### Statistical analysis

All analyses were performed using the Statistical Package for the Social Sciences (SPSS) Version 11.0 (SPSS Inc., Chicago, USA). Descriptive statistics (frequencies, percentages, means, and standard deviations [SD]) were first calculated. Subsequently, t-tests (for two-group comparisons) and analyses of variance (ANOVAs; for multi-group comparisons) were used to evaluate differences in continuous variables when the data were normally distributed and in accordance with assumptions regarding the homogeneity of variance. Otherwise, non-parametric methods were used (the Wilcoxon rank sum tests for two-group comparisons and the Kruskal-Wallis H tests for multi-group comparisons). All variables with *p* < 0.1 in the bivariate analysis were entered into the multivariate models. Multivariate liner regressions were used to identify independent risk factors for subjective health result. The entry and removal criteria for variables in the multivariate analysis were 0.05. A probability value of *p* < 0.05 was considered to be statistically significant.

## Results

### Physical examination results

Among the 190 study participants, 90 were male and 99 were female, with an average age of 12.8 ± 1.60 years old. The results of their physical examination are shown in Table 1. The average height was 142.27 ± 9.96, and the average weight was 34.38 ± 8.00. The BMI index ranged between 13.05-27.09, with the average being 16.8 ± 2.01. Among them, the average height of boys was 143.9 ± 10.32, their weight was 35.6 ± 8.31, and their BMI was 16.96 ± 2.08. The average height of girls was 140.73 ± 9.40, their average weight was 33.26 ± 7.57, and their average BMI was 16.58 ± 1.93. The average height and weight of boys and girls are statistically different (*p*-values of 0.026 and 0.044, respectively), but there is no statistically significant difference in BMI between boys and girls (p-value of 0.194).

**Table 1:** Results of Physical Examination

Category	Item	No.	%
Tooth missing	No	181	95.3
	Yes	9	4.7
Tooth decay	No	134	70.5
	Yes	56	29.5
Myopia	No	161	84.7
	Yes	29	15.3
Color blindness	No	188	98.9
	Yes	2	1.1
Smell disorders	No	187	98.4
	Yes	3	1.6
Throat problem	No	188	98.9
	Yes	2	1.1
Hearing problem	No	189	99.5
	Yes	1	0.5
Number of disorders in the physical exam	None	110	57.9
	One disorder	51	26.8
	Two disorders	21	11.1
	Three disorders	8	4.2
BMI Index	BMI < 18.5	165	86.8%
	18.5 ≤ BMI < 24	24	12.6%
	BMI ≥ 24	1	0.5%
Total		190	100%

### Information in five dimensions of the PROMIS questionnaire

**Fig. 1:**
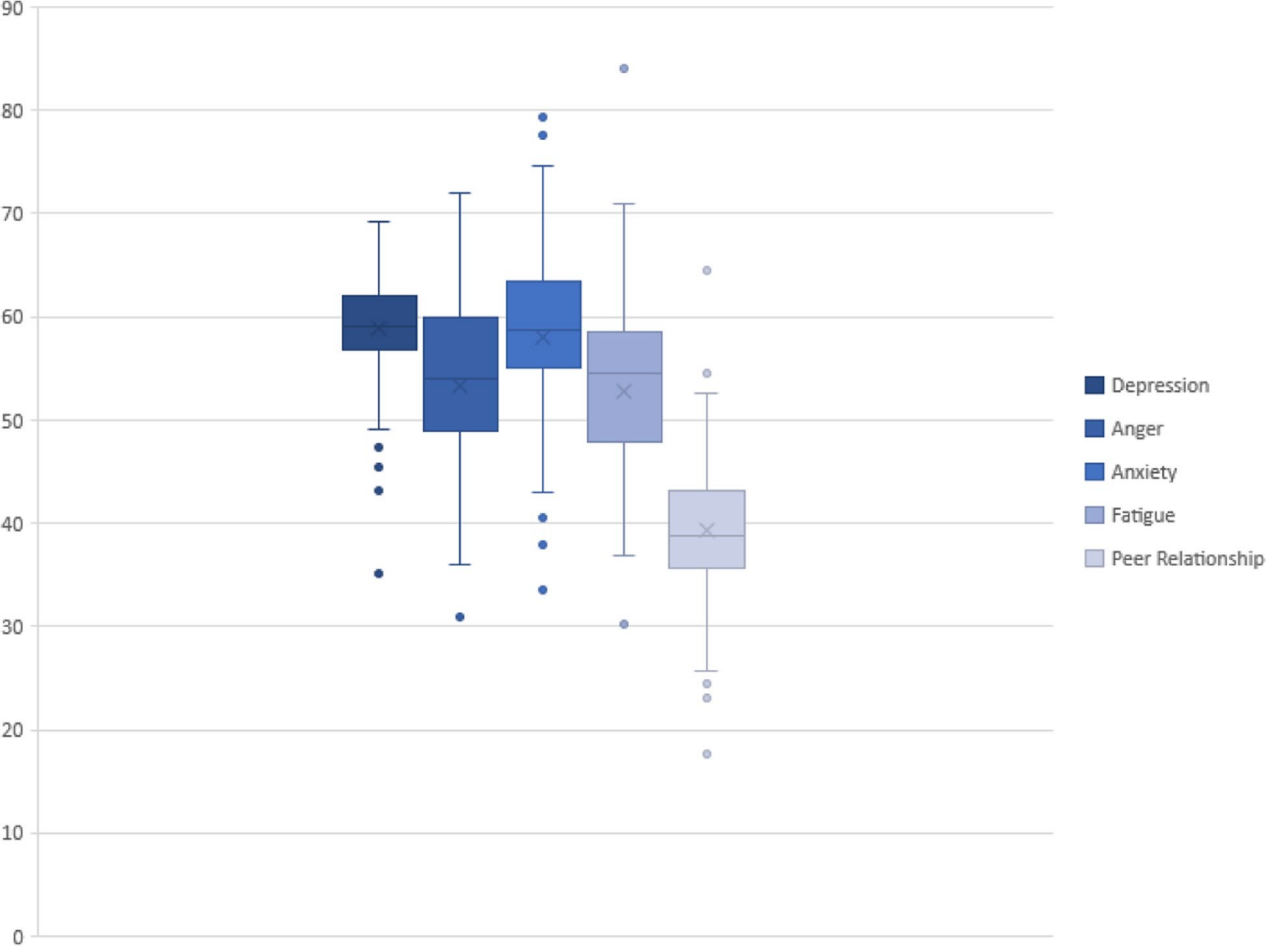
The five-dimensional health score box chart of the PROMIS

The average values of the five dimensions of depression, anger, anxiety, fatigue, and peer relationships in the PROMIS of the research subjects were 58.9 ± 5.3, 53.3 ± 8.0, 58.1 ± 7.3, 52.8 ± 8.0, 39.3 ± 6.6 (shown in Fig. 1). These are the same as the norms of the PROMIS population compared with 50 ± 10, and the *p*-values were all lower than 0.0001. The difference is statistically significant.

### Single-factor and multi-factor analysis between the health scores of the PROMIS and each factor

This study examined the correlation between the characteristics of each factor in the questionnaire and the five dimensions of the subjective health quality PROMIS scale. A single factor analysis was carried out on the three aspects of each questionnaire component: demographic information, family status, and school environment. The results are shown in Table 2. The indicators that were the most statistically significant derived from the single-factor analysis of the fatigue dimension (10 items), followed by the peer relationships dimension (7 items).

**Table 2:** Five health dimension scores and single-factor analysis results of each factor of the PROMIS

Item	Category	N	Percent %	p-value				
				Depression	Anger	Anxiety	Fatigue	Peer relationships
1. Demographic information and physical exam								
2003Grade	Grade 3	60	31.6	0.032	0.001	0.216	0.000	< 0.0001
	Grade 4	31	16.3					
	Grade 5	46	24.2					
	Grade 6	53	27.9					
Gender	Female	99	52.1	0.842	0.550	0.205	0.288	0.049
	Male	90	47.4					
Age	10	9	4.7	0.071	0.215	0.234	0.006	0.020
	11	26	13.7					
	12	59	31.1					
	13	37	19.5					
	14	28	14.7					
	15	21	11.1					
	> 15	10	5.3					
2. Family conditions								
Location	Urban	9	4.7	0.263	0.053	0.101	0.001	0.048
	Rural	115	60.5					
	Pastoral	60	31.6					
	Other	6	3.2					
Living with parents	Yes	178	93.7	0.896	0.903	0.469	0.997	0.148
	No	12	6.3					
The only child of the family	Yes	2	1.1	0.218	0.353	0.060	0.398	0.574
	No	188	98.9					
Father’s education level	Elementary school and below	73	38.4	0.271	0.295	0.053	0.011	0.039
	Middle school	10	5.3					
	High school and above	7	3.7					
	Don’t know	100	52.6					
Mother’s education level	Elementary and below	74	38.9	0.085	0.027	0.076	0.003	0.766
	Middle school	6	3.2					
	High school and above	7	3.7					
	Don’t know	103	54.2					
Family financial situation	Rich	19	10	0.023	0.105	0.004	0.031	0.276
	Middle level	151	79.5					
	poor	20	10.5					
3. School life								
No. of students in a single dormitory	Below 5	18	9.5	0.754	0.425	0.522	0.610	0.021
	5-10	39	20.5					
	11-15	111	58.4					
	above 15	22	11.6					
Visits home per month	4	119	62.6	0.978	0.682	0.299	0.275	0.387
	2-3	36	18.9					
	1	27	14.2					
	Sometimes not going home for one month	8	4.2					
Distance to home	Within 1 h	80	42.1	0.504	0.317	0.085	0.011	0.265
	2-4 h	81	42.6					
	4-6 h	22	11.6					
	6 h	7	3.7					
Relationship with schoolmates	Good	141	74.2	0.070	0.063	0.580	0.245	0.063
	General	46	24.2					
	bad	3	1.6					
Study pressure	Great	34	17.9	0.143	0.025	0.153	0.026	0.379
	General	133	70					
	Small or no pressure	23	12.1					
Hours of homework	Half an hour	72	37.9	0.731	0.203	0.033	0.001	0.692
	0.5-1 h	70	36.8					
	1-2 h	30	15.8					
	Above 2 h	18	9.5					
Hours of sleep each night	Above 10 h	24	12.6	0.574	0.673	0.487	0.042	0.049
	9-10 h	34	17.9					
	8-9 h	80	42.1					
	Below 8 h	52	27.4					
Total		190	100					

This study further incorporates the index of *p* < 0.10 in the single-factor analysis results into the multi-factor analysis. The results obtained are shown in Table 3. We found that in the demographic dimension, the grade was the main factor influencing anger (*p* < 0.01) and fatigue (*p* < 0.01), while gender was related to peer relationships (*p =* 0.02). In the family dimension, the father’s educational level was related to peer relationships (*p =* 0.05), while the family financial situation was related to depression (*p =* 0.01). In the school life dimension, relationship with classmates was found to affect anger (*p =* 0.05), while homework was related to anxiety (*p* = 0.02) and fatigue (*p =* 0.05).

**Table 3:** Multi-factor analysis results of the five health dimension scores in the PROMIS

category	Item	B	SE	p	95% CI interval
Depression	Family financial situation	2.11	0.83	0.01	0.47 ~ 3.74
Anger	Grade	2.02	0.46	< 0.01	1.11 ~ 2.932
	Relationship with classmates	2.26	1.15	0.05	−0.1 ~ 4.54
Anxiety	Homework burden	1.34	0.55	0.02	0.26 ~ 2.42
Fatigue	Grade	2.43	0.45	< 0.01	1.54 ~ 3.32
	Homework burden	1.14	0.56	0.05	0.02-2.25
Peer relationships	Sex	2.35	0.97	0.02	0.45 ~ 4.26
	Father education level	1.84	0.94	0.05	−0.14 ~ 3.69

## Discussion

In the field of adolescence studies, the PROMIS questionnaire has been widely used in the measurement of the subjective health status of children and adolescents with various chronic diseases, especially for children with cance r[15, 16, 25]. Subjective health evaluations are rarely carried out for ordinary adolescents, and few such studies conduct correlation analyses between subjective health evaluations and their objective physical health status. Our analysis of how Family involvement and School Life influence junior high school students’ subjective health can help conduct low-cost health tests for students. Such tests could encourage early health intervention measures for students and improving their growth environment. We found that the BMI of our study participants was generally lower than the BMI values ​​of Chinese students in other studies. The average BMI of adolescent aged seven to 18 in the study by Yun Wang is 20. 5[29], while the average BMI of students in grades seven to nine in the study by Miao Li increased from 18.2 to 20.1 from 2014 to 201 6[30]. In comparison, the BMI of the participants in our study was in the lean range. Our research participants belonged to ethnic minorities in underdeveloped areas of western China. American scholars have pointed out that ethnic minorities are vulnerable to discrimination, which has a negative impact on their mental and physical health as well as on their seeking of health service s[31, 32]. However, in China, policies promoting ethnic equality and even partial preference for ethnic minorities were implementin g[33]. Therefore, the health of the research participants was not related to ethnic discrimination but mainly affected by economic development. Most previous studies have found a correlation between the level of economic development and BMI. Therefore, it is necessary to improve the nutrition level of young people in economically underdeveloped areas. China has implemented a nutrition improvement plan for students in rural areas, who receive compulsory education at the national level. This is a means to accurately improve the health of young people in the context of limited economic developmen t[29]. The results of our study suggest that there is still room for improving the nutritional and health status of young people in China’s underdeveloped minority areas.

In the subjective health evaluation, there is currently no PROMIS score for the Chinese youth population. According to the official description of the PROMIS scale developed by the Health Measures team at Northwestern University, a PROMIS score of 50 is the average (or mean) score for a specific, relevant group of people (e.g., the US general population, children with a painful condition )[34]. We found that the scores for depression, anger, anxiety, and fatigue in the subjective health evaluation of our research participants were all higher than the norm, but the scores in the peer relationships were lower than the norm. According to the PROMIS scale evaluation standard, high scores in the depression, anger, anxiety, and fatigue dimensions resulted in a worsening of the health evaluation result. Additionally, a low lower the score in the peer relationships dimension resulted in a lower evaluation result. Our results indicate that the scores of the subjective health evaluation of ethnic minority students in Nangqian County of Yushu were lower than the norm. These findings are consistent with the physiological evaluation index of BMI, suggesting that attention should be paid to the improvement of the physical health, mental health, and social relationships of ethnic minority students in this region. Literature studies have found that PROMIS scores can be used to better judge the specific impact dimensions of HRQoL, which is conducive to precise interventions for specific dimensions. As an evaluation tool with high economic benefits, PROMIS can be used as an important means to carry out regular health assessments for students in the future because it can uncover key intervention targets in advance, which has been proven to have positive effects on chronic disease managemen t[35]. In terms of practical interventions, it is necessary to carry out health interventions from the key influencing factors based on the health assessment results. Therefore, it is particularly important to explore subjective factors influencing health.

The results of our study show that there were differences between male and female participants in the dimension of peer relationships, and that grade greatly affected the dimensions of anger and fatigue. We found that boys had better peer relationships than girls, which is supported by existing studies. Other scholars have found that girls are more likely to feel lonely, that their feelings are more sensitive, and that they are more susceptible to surrounding influences. This leads them to experience mood swings, which in turn affects their relationships with other s[1, 34]. Students who are marginalized or do not get along well with their peers may become victims of campus violence, or they may become perpetrators of campus violence. A study conducted in seven Chinese provinces also found that relationships with teachers, peer relationships, and academic performance can affect the occurrence of school bullyin g[36]. However, studies also demonstrate that campus violence is more likely to occur among boys, while campus violence among girls is more likely to be either relational or verba l[37–39]. In economically underdeveloped areas, the lack of educational resources is likely to lead to an increase in the frequency of school violence and bullyin g[40]. Such behaviors can seriously affect the mental health of students and can have a continuous impact on their behaviors in adulthoo d[2], which makes early intervention and treatment necessary. Therefore, we would recommend paying attention to the relationship between girls in their dormitories in order to foster healthy relationship between students and prevent the occurrence of campus bullying and violence.

As the students become older, the anger and fatigue evaluation results of their subjective health become worse. This may increase the stress levels of students. In China’s educational system, junior high school students are under greater learning pressure. Specifically, the pressure associated with the entrance examination in the third grade of junior high school can affect students’ future work choices and lives. With the development of China’s economy, parents’ attention to their adolescent’s learning has been further strengthened. Students in higher grades experienced more pressure, which had a greater impact on their mental health. The results of our study indicate that more attention needs to be paid to the irritability and fatigue manifested by students. The states of anger and fatigue can easily develop into depression, which can increase the risk of adolescent engaging in self-injurious behavior, including suicid e[41]. This can further damage the physical and mental health of students. At the school level, more psychological interventions should be available for senior students. The Ministry of Education of China has implemented relevant policies and issued various documents drawing attention to the mental health of school students and establishing a psychological consultation office in each school. Currently, in China, the mental health services available in schools located in economically developed areas are of high quality. However, psychological services in underdeveloped areas still needs to be further improved. Therefore, it is necessary for schoolteachers in underdeveloped areas, to pay attention to the mental health of senior students. They should focus on the manifestations of their fatigue, including sleepiness in class, poor concentration, and anger (for example, quarrels or even fights with classmates).

In addition, some studies have shown that religion can slow down the occurrence of symptoms of anger and other psychological-related disease s[42]. All our research participants were Tibetan students, but their subjective health performance in various dimensions was worse than normal. This may be related to the fact that young people are currently receiving more secular influences through various media, and they do not know much about religion or are less interested in religion. However, due to the rapid development of new media, there is no clear evidence of a direct relationship between youth media exposure and mental healt h[5, 14]. Further research could be carried out in this area of study.

In the analysis of family-related factors, we found that parents’ educational level and family economic status had an impact on students’ subjective health and peer relationships. We found that the education level of the father affected the peer relationship of students, which is closely related to the position of the parents in traditional Chinese culture. Fathers generally pay more attention to their children’s studies and their relations with the outside world, while mothers are more concerned about their children’s emotional stat e[43]. As a result, mothers are more likely to have an impact on the emotions of student s[44]. Previous studies have found that parents with a higher level of education were more likely to be considerate of their children’s emotions and play a stronger role in guiding their relationship with their classmates. After classmate conflicts occur, the intervention of schools and parents is very important. Regarding social adjustment and functioning, we found that students had better outcomes when the mother did not have a controlling parenting style and then the father had a controlling parenting styl e[45]. Therefore, for rural parents in underdeveloped areas, schools should educate them during the teacher-parent meeting on how to be mindful of their children’s emotional changes, how to strengthen their communication with children, and how to create a warmer family environment for the children.

Our results also indicated that the improvement of family economic status led to the alleviation of depression among students. Previous studies have repeatedly found that family conditions affect the physical and mental development of student s[46–48]. Moreover, economic conditions affect the family’s access to education and other important information, which in turn affects students’ mental healt h[49, 50]. In addition, a family’s poor economic situation also affects the psychological pressure of parents and has a further negative impact on their adolescent. Therefore, schools should pay more attention to students with average family conditions. Previous studies have also demonstrated that, in addition to culture and economy, the family environment affects the peer relationships and physical and depression symptoms of student s[51]. Adverse mental conditions will, in turn, affect the family life of students and reduce their quality of lif e[52, 53]. Current research shows that the occurrence of depression is mostly related to students’ negative life experiences or poor family environment. There is no literature showing that the mental health of students is related to their life experience or their cognitive social experienc e[49]. Therefore, it is recommended to further strengthen the care of students with average family economic conditions. These include increasing home visits, setting up psychological consultation offices at school, and other measures so that schools can understand their students’ family environment during their growing up period, take appropriate measures to protect students from negative life experience, and ensure their students’ physical and mental health.

School life will have an impact on the subjective health of students, and more homework will lead to anxiety and fatigue. In China, most of the education at school is exam-oriented, which causes competition among students. Therefore, many schools aim to increase students’ knowledge through constant homework and repeated practice, which requires them to do a lot of homework after schoo l[54]. Studies have found that excessive homework may lead to a lack of sleep for students, which may cause a variety of negative effects, including poor concentration and poor academic performance. These negative behaviors will lead to the deterioration of students’ mental healt h[55]. Our research also demonstrates that an unreasonable amount of homework adversely affects the physical and mental development of students. Julius Ohrnberger and others have analyzed the relationship between the mental and physical health of 10,693 people over 10 years and found that mental state can lead to changes in physical state after a long period of tim e[30]. The resulting changes in health status may even be permanent. For students, their anxiety and fatigue will further affect their family relationships and life, and further produce changes in their physical development and disease status. Therefore, it is suggested that schools should assign homework in moderation, coordinate the amount of homework in each school subject, and enable students to have sufficient sleep hours.

## Conclusion

For ethnic minority areas that are relatively underdeveloped in the western part of China, the physical health index BMI and subjective health evaluation of students are worse than those in developed areas. Their family environment and school life all have varying degrees of impact on the five subjective health outcomes. There are differences in gender and grade level. The government and society need to pay more attention to the physical and mental health of students in remote and underdeveloped areas and improve their health through a student nutrition plan and the establishment of mental health offices. School teachers not only need to care about students’ achievements, but also about their students’ mental health. Teachers should try to spot unfavorable emotional and growth stunting issues in students at an early stage, and work with their families as soon as possible to improve the level of health.

## Data Availability

The datasets used and/or analysed during the current study are available from the corresponding author on reasonable request.

## Abbreviations

PROMIS: The Pediatric Patient-Reported Outcomes Measurement Information System
HRQoL: Health related quality of life
QoL: Quality of life
PCS: Physical component summary scale
MCS: Mental component summary scale
BMI: Body Mass Index
